# Vertrauen der Bevölkerung in staatliche Institutionen im ersten Halbjahr der Coronapandemie: Erkenntnisse aus dem Projekt COVID-19 Snapshot Monitoring (COSMO)

**DOI:** 10.1007/s00103-021-03279-z

**Published:** 2021-01-29

**Authors:** Sarah Eitze, Lisa Felgendreff, Lars Korn, Philipp Sprengholz, Jennifer Allen, Miriam A. Jenny, Lothar H. Wieler, Heidrun Thaiss, Freia De Bock, Cornelia Betsch

**Affiliations:** 1 CEREB – Center of Empirical Research in Economics and Behavioral Sciences, Media and Communication Science, Universität Erfurt, Nordhäuser Str. 63, 99089 Erfurt, Deutschland; 2 Universität Erfurt, Erfurt, Deutschland; 3 Robert Koch-Institut, Berlin, Deutschland; 4 Max-Planck-Institut für Bildungsforschung, Berlin, Deutschland; 5 Harding-Zentrum für Risikokompetenz, Universität Potsdam, Potsdam, Deutschland; 6 Bundeszentrale für gesundheitliche Aufklärung, Köln, Deutschland

**Keywords:** Gesundheitspsychologie, Vertrauen in Institutionen, Risikowahrnehmung, Krisenkommunikation, Coronaviruspandemie, Health psychology, Trust in institutions, Affective risk, Crisis communication, COVID-19 pandemic

## Abstract

**Hintergrund:**

In der Coronaviruspandemie nehmen 2 Institutionen eine zentrale Rolle in der evidenzbasierten Einordnung des Geschehens für Politik und Bevölkerung ein. Das Robert Koch-Institut (RKI) koordiniert die Pandemiebekämpfung, erstellt fundierte Empfehlungen für medizinisches Fachpersonal, die Medien sowie die Bevölkerung und berät die Politik. Die Bundeszentrale für gesundheitliche Aufklärung (BZgA) informiert die Bevölkerung und Institutionen.

**Ziel der Arbeit:**

Mit dem COVID-19 Snapshot Monitoring (COSMO) wird beobachtet, ob und wie sich das Vertrauen in Institutionen über die Pandemie verändert. Es wird untersucht, welche Bevölkerungsgruppen Vertrauen zeigen und wie dies mit Einstellungen, Risikowahrnehmung und Verhaltensweisen zusammenhängt.

**Material und Methoden:**

In Querschnittstudien werden seit März 2020 die Risikowahrnehmung, das Verhalten und die Akzeptanz von Maßnahmen sowie das Vertrauen in Institutionen mit etwa *N* = 1000 Befragten pro Erhebung untersucht.

**Ergebnisse:**

Das Vertrauen in RKI und BZgA war generell hoch, sank aber über den Verlauf der Pandemie. Höheres Vertrauen ging für beide Institutionen mit höherem Alter der Befragten, höherer Bildung, höherer Risikowahrnehmung und höherer Akzeptanz von Maßnahmen einher.

Verhaltensweisen wie Abstandhalten und Händewaschen wurden häufiger gezeigt. Männer und chronisch Erkrankte zeigten geringeres Vertrauen.

**Diskussion:**

Die Ergebnisse zeigen, dass Vertrauen weiter gefördert werden sollte. Dies könnte u. a. erreicht werden, indem in der Entwicklung und Begründung von Strategien und Maßnahmen auch die Sichtweise der Bevölkerung (z. B. durch COSMO) berücksichtigt wird. Kommunikationsstrategien und Handlungsempfehlungen sollten darauf abzielen, Personen mit hoher Risikowahrnehmung zu unterstützen und zu entlasten.

## Abstract (en)

**Background:**

In the coronavirus pandemic, two institutions play a central role in the evidence-based classification of events for politics and the population. The Robert Koch Institute (RKI) coordinates the fight against the pandemic, prepares well-founded recommendations for medical professionals, the media and the population, and advises politicians. The Federal Centre for Health Education (BZgA) informs the population and institutions.

**Goals:**

The COVID-19 Snapshot Monitoring (COSMO) project monitors whether and how trust in institutions changes over the pandemic. Which population groups show trust and how this is related to attitudes, risk perception and behaviour are analysed.

**Methods:**

Cross-sectional studies with approximately *N* = 1000 respondents per survey were conducted since March 2020 to investigate risk perception, behaviour, acceptance of measures and trust in institutions.

**Results:**

Trust in the RKI and BZgA was generally high but declined over the course of the pandemic. Higher trust for both institutions was associated with higher age of respondents, higher education, higher risk perception and higher acceptance of measures. Behaviours such as physical distancing and handwashing were shown more frequently. Men and the chronically ill showed lower trust.

**Discussion:**

The results show that trust should be further promoted. This could be achieved, among other things, by taking into account the population’s perspective (e.g. through COSMO) in the development and justification of strategies and measures. Communication strategies and recommendations for action should aim to support and relieve people with high-risk perceptions.

## Hintergrund

Die COVID-19-Pandemie ist die bisher größte gesundheitspolitische Herausforderung unseres Jahrhunderts [1, 2]. Die internationale Vernetzung trägt sowohl zu einer umfangreichen Berichterstattung als auch einem schnellen Erkenntnisgewinn über die neuartige Krankheit durch die Wissenschaft bei. Trotzdem steht rund 9 Monate nach Beginn der Pandemie keine ausreichend effektive pharmakologische Intervention, sondern ausschließlich die Verhaltensänderung der Bevölkerung als effektive Bewältigungsstrategie zur Verfügung [3].

Innerhalb der ersten Monate der Pandemie wurden die täglichen Pressebriefings des Robert Koch-Instituts (RKI) in der Presse zitiert und auch live übertragen. Parallel dazu baute die Bundeszentrale für gesundheitliche Aufklärung (BZgA) ein strukturiertes Informationsangebot für die breite Bevölkerung auf, welches Verhaltensempfehlungen und die aktuelle Sachlage in diversen Sprachen und auch in leichter Sprache oder für weitere spezifische Bedarfe anbietet. Die Gesundheitsinformationen wurden zudem zielgruppengerecht für Familien, Einrichtungen (wie Kitas und Schulen) sowie für Arbeitgeber, Pflegekräfte und Minoritäten aufbereitet. Für diese Informationsangebote an Politik und Bevölkerung leiten das RKI und die BZgA aus der aktuellen Evidenz die bestmöglichen Handlungsempfehlungen ab. Ob ein ausreichend großer Anteil der Bevölkerung diesen Empfehlungen folgt, hängt – basierend auf Erkenntnissen aus früheren Pandemien und Krisensituationen – unter anderem vom Vertrauen in diese beiden Institutionen ab [1].

Eine Erwartung in Aufrichtigkeit und Wahrhaftigkeit der Institutionen [4] sowie die Wahrnehmung der Institution als wohlwollend [5], moralisch und rechtlich den Bürger/innen verpflichtet [6] bestimmt das Vertrauen. Vertrauen in Institutionen wird als kognitive Einstellung verstanden, die vertrauende Handlungen (die Einhaltung von gesetzlich erlassenen Maßnahmen und darüber hinausgehenden Empfehlungen) motivieren kann [7]. Daher sollte das Ausmaß an Vertrauen in die Institutionen dazu beitragen, wie stark die Bevölkerung Hygienemaßnahmen, Abstandsregeln und das Tragen von Gesichtsmasken in ihr pandemisches Verhaltensmuster aufnimmt.

Das Vertrauen in Institutionen beeinflusst die Risikowahrnehmung (reduziert Emotionen wie Angst, Sorge, Stress [8]), hilft beim Umgang mit Komplexität und ist ausschlaggebend für die Legitimierung von Behördenentscheidungen [9]. Damit ist Vertrauen in die Institutionen ein relevanter Einflussfaktor auf die Entwicklung der Pandemie [10]. Da Vertrauen durch eigene Erfahrungen und durch die Erfahrungen wichtiger, stellvertretender Personen (erlebbar z. B. durch Berichte in den Medien) generalisiert wird [11], müssen Veränderungen im Vertrauen über den Verlauf der Pandemie mit dem Vertrauen in Institutionen vor der Pandemiesituation verglichen werden.

Obwohl Vertrauen in Institutionen seltener untersucht wird als Vertrauen zwischen Personen [12], stehen einige Studien als Vergleichswerte aus Zeiten vor der Pandemie zur Verfügung. Daten aus den ALLBUS-Studien zeigen, dass etwa 2 Drittel der Deutschen ein Mindestvertrauen (mittleres bis hohes Vertrauen) in das deutsche Gesundheitssystem haben [12]. Trotzdem ist über die Jahre ein Vertrauensverlust erkennbar. Im Gesundheitsmonitor der Bertelsmann-Stiftung haben über 50 % der Befragten ein mittleres bis hohes Vertrauen in das Gesundheitssystem angegeben [13]. Allerdings gibt nur ein geringer Teil der Probanden hohes Vertrauen an, wenn nach dem Vertrauen in spezifische Institutionen wie dem Bundesministerium für Gesundheit (19 %) oder die Regierung (25 %) gefragt wird. Weiter zeigt sich, dass sich das Vertrauen zwischen verschiedenen Zielgruppen stark unterscheidet. So haben z. B. 72 % der höher gebildeten Probanden angegeben, ein höheres Vertrauen in das Gesundheitssystem zu haben, während dies nur für 28 % der Probanden mit niedriger Bildung gilt [14].

Ziel dieser Arbeit ist es, aus den querschnittlichen Daten der COVID-19 Snapshot Monitoring(COSMO)-Erhebungen über den Verlauf des ersten halben Jahres der Coronaviruspandemie zu explorieren, ob und ggf. wie sich das Vertrauen in die 2 Institutionen im Verlauf der ersten 6 Monate der Pandemie geändert hat und wie es mit pandemiespezifischen Einstellungen, der affektiven Risikowahrnehmung und Verhaltensweisen zusammenhängt. Zudem sollen Gruppen identifiziert werden, die von Interventionen zur Steigerung von Vertrauen profitieren könnten.

## Methode

### Prozedur

In querschnittlichen Onlinebefragungen wurden mit Beginn der Pandemie in Deutschland seit dem 03.03.2020 wöchentlich etwa 1000 Personen befragt [15]. Ab dem 26.05.2020 erfolgte die Erhebung zweiwöchentlich. Die Stichproben wurden gemäß des Zensus 2011 [16] nach Alter, Geschlecht (gekreuzt) und Bundesland (ungekreuzt) der deutschen Bevölkerung quotiert. Eine Erhebungsphase dauerte jeweils von 10 Uhr morgens am Dienstag bis 24 Uhr am darauffolgenden Mittwoch, umfasst also ca. 2 Tage.

### Material

Rohdaten, eine Übersicht aller verwendeten Variablen sowie das Durchführungsskript der Analysen sind online verfügbar [17]. Als soziodemografische Variablen wurden Alter, Geschlecht, Bildung (niedrig: weniger als 9 Jahre, mittel: > 10 Jahre ohne Abitur, hoch: > 10 Jahre mit Abitur) sowie Wohnortgröße (Kleinstadt: < 5000 Einwohner, Mittelstadt: > 5000–100.000 Einwohner, Großstadt: > 100.000 Einwohner) erhoben. Die Auswahl dieser soziodemografischen Variablen unterliegt sowohl theoretischen Überlegungen (mögliches *Targeting *von vertrauensfördernden Maßnahmen [18, 19]) als auch methodischen Überlegungen (durchgängig erhobene Variablen über alle Erhebungszeitpunkte, Begrenzung der Prädiktoren insgesamt). Vertrauen wurde ab der ersten Erhebung auf einer 7‑stufigen Skala (1 sehr wenig Vertrauen bis 7 sehr viel Vertrauen) erhoben [15]. In randomisierter Reihenfolge wurde Vertrauen in das RKI, die BZgA sowie in andere Institutionen, wie z. B. die Regierung, die Gesundheitsministerien oder Ärzte und Krankenhäuser, erhoben.

Akzeptanz der Maßnahmen wurde mit 2 Items ab der 10. Erhebung (05.05.2020) abgefragt. Jeweils auf einer 7‑stufigen Skala gaben die Teilnehmenden an, ob (a) die Einschränkungen und (b) die Lockerungen der Einschränkungen als übertrieben wahrgenommen werden (1 stimme überhaupt nicht zu bis 7 stimme voll und ganz zu).

Verhalten wurde ab der 7. Erhebung (14.04.2020) 5‑stufig abgefragt (1 nie, 2 selten, 3 manchmal, 4 häufig, 5 immer). Beim Verhalten konnte man „trifft nicht zu“ auswählen. Diesen Teilnehmenden werden dann fehlende Werte zugeordnet, sie gehen nicht in die Analysen mit ein. Verhalten zur AHA+L-Regel (Abstand wahren, auf Hygiene achten, Alltagsmaske tragen, Lüften) wird in einem Mittelwert zusammengefasst (Cronbachs Alpha: 0,57). Außerdem wird die Tendenz, private Feiern sowie öffentliche Orte zu meiden, untersucht.

Affektive Reaktionen wurden mithilfe semantischer Differenziale ab der 7. Erhebung (14.04.2020) ohne Zeitbezug erfasst („Das neuartige Coronavirus ist für mich …“). Teilnehmende orteten sich in einem 7‑stufigen Bereich zwischen 2 Endpunkten ein (z. B. angsteinflößend – nicht angsteinflößend). Die Einschätzungen zu Angst, Besorgnis und Präsenz des Themas (umcodiert in 1 niedrig bis 7 hoch) werden in einem Mittelwert zu negativem Affekt zusammengefasst (Cronbachs Alpha: 0,82). Dieser wird als Indikator der affektiven Risikowahrnehmung interpretiert [20].

Weitere Bereiche (z. B. Wissen oder Lebenszufriedenheit) werden ebenfalls regelmäßig abgefragt. Zu den Interessenschwerpunkten einzelner Erhebungen zählen psychologische Konstrukte, wie Resilienz oder Wohlbefinden, und experimentelle Studiendesigns zu (potenziellen) eindämmenden Maßnahmen [21]. Die Erhebungen dauern durchschnittlich zwischen 15 min und 20 min. Das COSMO-Projekt wurde vom Ethikbeirat der Universität Erfurt geprüft und als ethisch unbedenklich bewertet (#20200501).

### Auswertung

Im ersten Schritt werden die absoluten Vertrauenswerte gegenüber den Institutionen RKI und BZgA in Bezug zu denen der anderen Institutionen gesetzt. Anschließend wird für die beiden Institutionen anhand linearer Regressionen mit Rückwärtselimination ermittelt, ob bestimmte Bevölkerungsgruppen (Alter, Geschlecht, Bildung, Wohnortgröße, Beruf im Gesundheitssektor, chronische Erkrankung) stärkeres oder schwächeres Vertrauen zeigen sowie ob sich dieser Effekt über die Zeit verändert (als Interaktionsterm für Zeit × demografischer Prädiktor). Zuletzt wird mit bivariaten Korrelationen geprüft, ob Vertrauen in die Institutionen mit affektiver Risikowahrnehmung, Akzeptanz von Maßnahmen und Verhalten zusammenhängt. Damit lässt sich die Frage beantworten, ob Personen mit höherem Vertrauen in Institutionen die Pandemiesituation besser bewältigen und stärker zur gemeinsamen Bekämpfung der Pandemie durch Verhaltensveränderung beitragen.

## Ergebnisse

Insgesamt gingen *N* = 18.874 Befragte aus 19 Erhebungen in die Auswertung ein. Eine genaue demografische Verteilung kann Tab. 1 entnommen werden. Durch fehlende Werte wurden die unstandardisierten Regressionen mit *N* = 16.109 Befragten durchgeführt. In den Korrelationen zwischen Vertrauen in das RKI und die BZgA und Akzeptanz (*N* = 9689), Risikowahrnehmung (*N* = 12.409) und Verhalten (*N* = 12.409) werden Teilnehmer/innen ab der 7. (14.04.2020) bzw. 10. (26.05.2020) Erhebung berücksichtigt.

**Table 1:** 

		Erhebungszeitpunkte 2020																		
	Total	03.03.	10.03.	17.03.	24.03.	31.03.	07.04.	14.04.	21.04.	28.04.	05.05.	12.05.	19.05.	26.05.	09.06.	23.06.	07.07.	21.07.	04.08.	18.08.
*Alter*																				
18–29	3596	190	181	196	189	199	175	207	169	211	196	192	188	192	165	178	187	194	192	195
30–49	7069	350	348	393	349	396	401	386	392	357	390	378	391	335	372	387	380	361	373	330
50–64	5276	275	283	275	266	278	287	262	295	290	274	279	273	273	282	272	281	278	277	276
65–74	2933	162	157	154	153	157	161	179	156	162	147	165	120	125	136	156	162	168	157	156
Total	18.874	977	969	1018	957	1030	1024	1034	1012	1020	1007	1014	972	925	955	993	1010	1001	999	957
*Geschlecht*																				
Männlich	9291	493	462	507	495	507	507	504	491	488	503	493	477	441	464	483	494	490	496	496
Weiblich	9583	484	507	511	462	523	517	530	521	532	504	521	495	484	491	510	516	511	503	461
Total	18.874	977	969	1018	957	1030	1024	1034	1012	1020	1007	1014	972	925	955	993	1010	1001	999	957
*Bundesland*																				
B.-W.	2274	120	121	131	118	129	129	129	128	128	105	108	107	114	114	129	130	117	126	91
Bayern	2842	137	130	157	160	158	155	159	156	153	157	161	146	133	145	146	151	130	156	152
Berlin	838	43	44	44	34	46	45	44	48	44	46	48	44	44	43	43	46	45	44	43
Brandenburg	586	31	32	33	26	32	30	34	21	33	32	32	32	30	27	34	30	31	33	33
Bremen	157	8	8	8	8	9	9	8	10	9	8	9	7	8	7	7	8	9	9	8
Hamburg	442	22	23	23	22	24	22	22	27	22	25	23	22	23	21	22	25	26	22	26
Hessen	1405	71	68	70	70	75	76	76	76	75	78	76	73	71	76	72	74	78	75	75
Meckl.-Vorp.	393	25	19	21	23	27	21	21	21	21	23	18	22	15	17	20	21	22	15	21
Nieders.	1797	92	92	95	96	96	104	97	95	98	97	99	95	72	92	96	95	98	96	92
NRW	4163	219	218	227	187	217	219	230	223	222	227	222	227	210	208	213	221	233	217	223
RLP	966	51	50	49	58	55	55	53	51	49	50	51	49	48	50	52	49	54	45	47
Saarland	244	15	15	16	13	14	10	11	14	16	13	9	13	13	12	13	13	9	15	10
Sachsen	1002	50	55	52	45	52	54	56	62	53	51	59	51	53	48	51	50	53	56	51
S‑Anhalt	548	31	30	29	35	31	30	29	26	32	31	30	25	27	29	29	30	26	27	21
Schleswig‑H	684	36	36	36	30	35	36	37	35	37	36	41	34	36	35	35	37	40	36	36
Thüringen	533	26	28	27	32	30	29	28	19	28	28	28	25	28	31	31	30	30	27	28
Total	18.874	977	969	1018	957	1030	1024	1034	1012	1020	1007	1014	972	925	955	993	1010	1001	999	957
*Schulbildung*																				
Bis zu 9 Jahre	2062	107	103	122	96	104	90	128	114	112	95	110	105	94	96	112	107	130	123	114
>10 Jahre (ohne Abitur)	6387	352	337	376	326	360	354	327	329	353	319	328	334	301	353	340	362	324	299	313
>10 Jahre (mit Abitur)	10.425	518	529	520	535	566	580	579	569	555	593	576	533	530	506	541	541	547	577	530
Total	18.874	977	969	1018	957	1030	1024	1034	1012	1020	1007	1014	972	925	955	993	1010	1001	999	957

Abgekürzte Bundesländer: *B.W.* Baden-Württemberg, *Meckl.-Vorp.* Mecklenburg-Vorpommern, *Nieders.* Niedersachsen, *NRW* Nordrhein-Westfalen, *RLP* Rheinland-Pfalz, *S‑Anhalt* Sachsen-Anhalt, *Schleswig‑H* Schleswig-Holstein

### Vertrauen in der Pandemie

**Figure 1:**
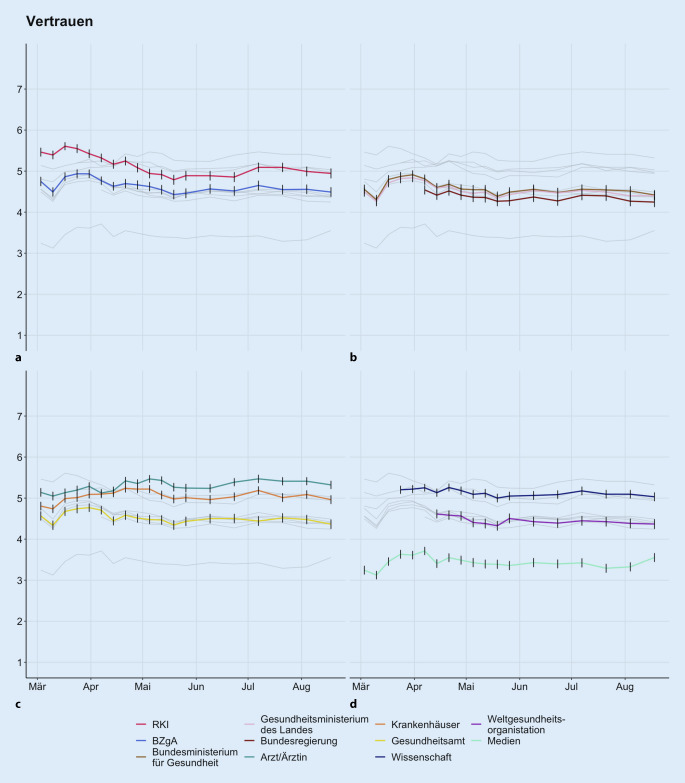

Abb. 1 zeigt die Selbstauskunft zum Vertrauen in die Institutionen über die Zeit und erlaubt einen Vergleich zwischen RKI und BZgA (a) sowie zwischen verschiedenen in der Krise verantwortlichen politischen Organen (Gesundheitsministerium, Bundesregierung (b)), dem Gesundheitswesen (Gesundheitsämter, Krankenhäuser, Ärzte (c)) und der Wissenschaft, der Weltgesundheitsorganisation (WHO) und den Medien (d). Das RKI hat gemeinsam mit der Wissenschaft die höchsten Vertrauenswerte. Die BZgA verortet sich auf gleicher Ebene mit politischen Organen. Insgesamt ist das Vertrauen in die Verantwortlichen in der Krise hoch und über die Zeit relativ stabil. Besonders in der Phase der Kontaktbeschränkungen zu Beginn der Pandemie (Ende März–Anfang Mai) war das Vertrauen hoch. Den Medien wird insgesamt am wenigsten vertraut – sie liegen konstant unter dem Skalenmittelpunkt (Wert 4), der Indifferenz ausdrückt.

### Vertrauen in das Robert Koch-Institut

Das Vertrauen in das RKI war während der Anfangsphase der Pandemie sehr hoch und sank über die Dauer der Pandemie leicht (Abb. 1; Tab. 2: Prädiktor „Erhebungszeitpunkt“). Ein höheres Vertrauen in das RKI geht über alle Erhebungszeitpunkte mit höherem Alter der Befragten, höherem Bildungsniveau, Vorliegen einer chronischen Erkrankung und höherer Einwohnerzahl der eigenen Gemeinde einher. Befragte Männer weisen signifikant weniger Vertrauen in das RKI auf als Frauen. Die Interaktionseffekte zwischen Zeit und Geschlecht zeigen allerdings, dass dieser Unterschied im Zeitverlauf verschwindet, weil Frauen deutlicher an Vertrauen verlieren. Vertrauen in das RKI sinkt in kleinen Gemeinden deutlicher als in großen.

**Table 2:** 

	Vertrauen in das RKI			Vertrauen in die BZgA		
Variablen	*B*	95 %-KI	*p*	*B*	95 %-KI	*p*
Erhebungszeitpunkt	−0,04	−0,05–−0,03	**<0,001**	−0,02	−0,04–−0,01	**0,003**
Alter	0,01	0,01–0,01	**<0,001**	0,00	0,00–0,01	**<0,001**
Geschlecht: weiblich (vs. männlich)	0,16	0,10–0,21	**<0,001**	0,14	0,09–0,19	**<0,001**
Schulbildung: > 10 Jahre ohne Abitur (vs. 9 Jahre)	0,09	−0,01–0,18	0,075	0,07	−0,02–0,16	0,114
Schulbildung: Abitur (vs. 9 Jahre)	0,32	0,22–0,41	**<0,001**	0,26	0,17–0,35	**<0,001**
Chronisch krank vs. nicht chronisch krank	0,07	0,01–0,13	**0,017**	0,04	−0,01–0,10	0,141
Beruf im Gesundheitssektor	–	–	–	−0,07	−0,16–0,02	0,134
Wohnort: Mittelstadt (vs. Kleinstadt)	0,05	−0,02–0,12	0,156	0,05	−0,02–0,11	0,165
Wohnort: Großstadt (vs. Kleinstadt)	0,08	0,02–0,14	**0,011**	0,10	0,04–0,16	**0,001**
Interaktion Zeit × weibl. Geschlecht	−0,01	−0,02–−0,00	**0,008**	−0,02	−0,03–−0,01	**0,002**
Interaktion Zeit × Schulbildung: > 10 Jahre ohne Abitur (vs. 9 Jahre)	0,01	−0,00–0,02	0,124	–	–	–
Interaktion Zeit × Schulbildung: Abitur (vs. 9 Jahre)	0,02	0,00–0,03	**0,012**	–	–	–
Interaktion Zeit × Wohnort: Mittelstadt (vs. Kleinstadt)	–	–	–	0,01	−0,01–0,03	0,265
Interaktion Zeit × Wohnort: Großstadt (vs. Kleinstadt)	–	–	–	0,02	0,00–0,04	**0,021**
*N*	16.109			16.109		
R^2^/korrigiertes R^2^	0,026/0,026			0,011/0,011		

Ergebnisse der 2 multiplen Regressionen für Vertrauen in RKI und BZgA mit schrittweiser Rückwärtselimination von Prädiktoren, die nicht zur Varianzaufklärung beitragen. Prädiktoren im Modell: Zeit, Alter, Geschlecht, Bildung, Wohnortgröße, Beruf im Gesundheitssektor, chronische Erkrankung; Interaktionsterm Zeit × demografischer Prädiktor. Es werden unstandardisierte Regressionsgewichte und deren 95 %-Konfidenzintervalle gezeigt. Die hervorgehobenen *p*-Werte sind statistisch signifikant

### Vertrauen in die BZgA

Auch das Vertrauen in die BZgA sinkt leicht über die Zeit (Abb. 1). Und auch für höheres Vertrauen in die BZgA sind höheres Alter, höherer Bildungsgrad und höhere Einwohnerzahl der Gemeinde fördernde Prädiktoren (Tab. 2). Frauen haben höheres Vertrauen in die BZgA als Männer. Die Interaktionseffekte zwischen Zeit und Bildung zeigen, dass höher gebildete Menschen nicht nur generell höheres Vertrauen in die BZgA haben, sondern dies über die Erhebungen hinweg auch eher aufrechterhalten. Teilnehmende mit niedriger Bildung starten mit geringerem Vertrauen und verlieren Vertrauen über die Zeit. Die Effektstärken fallen in dieser Analyse insgesamt geringer aus als beim Vertrauen in das RKI.

Über beide Regressionen leisten die demografischen Variablen insgesamt nur eine geringe Aufklärung von Unterschieden im Vertrauen (RKI r^2^ = 0,026, BZgA r^2^ = 0,011, Tab. 2).

### Welche pandemierelevanten Variablen hängen mit Vertrauen zusammen?

**Figure 2:**
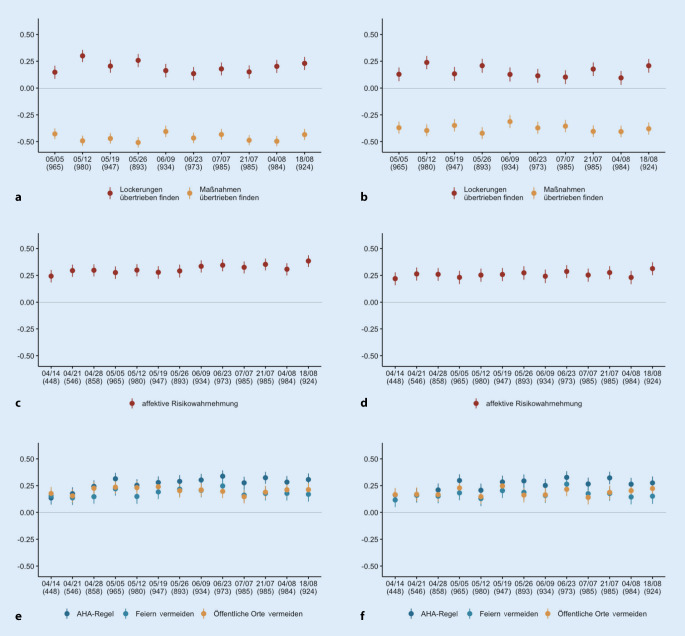

Abb. 2 zeigt Korrelationen zwischen pandemierelevanten Variablen und Vertrauen. Über alle Erhebungen zeigen sich signifikante Zusammenhänge zwischen dem Vertrauen in Institutionen und der Einschätzung von Maßnahmen und Lockerungen (a, b), der affektiven Risikowahrnehmung (c, d) sowie Verhalten (e, f). Die Korrelationen und Zeitverläufe sind für BZgA und RKI nahezu identisch. Für diese Analysen liegen vollständige Daten aus 10 Erhebungen (Mai 2020–August 2020) vor. Vertrauen und Verhalten korrelieren positiv: Wer den Institutionen mehr vertraut, befolgt auch eher die Empfehlungen zur AHA+L-Regel, zum Vermeiden von Feiern und zur Vermeidung von öffentlichen Orten. Die Höhe der Korrelation ist für die freiwilligen Maßnahmen (öffentliche Orte und Feiern vermeiden) über die Befragungen stabil, während der Zusammenhang für das Einhalten der AHA-Regel sogar leicht steigt. Die affektive Risikowahrnehmung ist höher, wenn das Vertrauen in die Institutionen auch höher ist. Teilnehmende, die den Institutionen mehr vertrauen, finden Lockerungen eher übertrieben und Maßnahmen eher nicht übertrieben.

## Diskussion

Das Vertrauen in die Institutionen, die die Bevölkerung und Politik mit evidenzbasierten Informationen versorgen, ist hoch ausgeprägt und über die ersten 6 Monate der COVID-19-Pandemie relativ stabil. Es gibt geringe Vertrauensverluste über die Dauer der Pandemie. In Bezug auf Bevölkerungsmerkmale wie Alter, Geschlecht oder Gemeindegröße bestehen ebenfalls nur geringfügige Unterschiede, wie die Effektgrößen und Gesamtvarianz der Regressionen zeigen. Bildung weist über die Zeit einen (wenn auch geringen) schützenden Effekt gegen Vertrauensverlust auf.

Wer den Institutionen vertraut, hält sich eher an die Regeln und die Einstellung zu Maßnahmen ist weniger ablehnend. Hohe affektive Risikowahrnehmung und hohes Vertrauen in die Institutionen hängen zusammen: Wer dem RKI und der BZgA mehr vertraut, nimmt die Pandemie als ein größeres Risiko wahr (und umgekehrt). Auch wenn hier die Kausalrichtung nicht festzustellen ist, können Personen mit einer hohen affektiven Risikowahrnehmung als eine wichtige Zielgruppe identifiziert werden, da sie offensichtlich gut durch die Gesundheitsbehörden erreichbar sind. So sollten Behörden diesen nicht nur konkrete Handlungsempfehlungen zur Risikoreduktion anbieten, sondern auch Informationen zur Bewältigung eventueller psychologischer Belastungen, die bei Personen mit hoher affektiver Risikowahrnehmung ebenfalls höher sind [22].

Stabiles Vertrauen in Institutionen in und zwischen Pandemiephasen ist für die erfolgreiche Bewältigung der Pandemie von hoher Bedeutung [1]. Da Vertrauen fluktuiert (Abb. 1) und auch über die Zeit unterschiedlich stark mit der Akzeptanz der Maßnahmen zusammenhängt (Abb. 2a, b), sollte es regelmäßig durch Umfragen erfasst [10] und bei Bedarf durch Interventionen adressiert werden [23]. Die Ursache der Fluktuation über die Zeit könnte sich in den Umstellungen der Maßnahmen finden: Im Entscheidungsprozess für die Lockerungen der Maßnahmen wurden zuerst Epidemiologen des RKI und Virologen, im Verlauf dann auch Wirtschaftswissenschaftler, Bildungsexperten und Experten anderer Sachgebiete zur Entscheidungsfindung herangezogen, die nicht als Stellvertreter für die Institutionen RKI oder BZgA sprechen. Die Maßnahmen und Lockerungen waren dabei jedoch immer Entscheidungen unter Unsicherheit. Auch im weiteren antizipierten Verlauf dieser Pandemie werden Entscheidungen unter Unsicherheit zu treffen sein. Ein hohes Vertrauen in die Institutionen, die die Politik und die Bevölkerung direkt in Entscheidungen beraten, kann die Akzeptanz der Entscheidungen selbst fördern [9].

Möglichkeiten, das Vertrauen weiter zu steigern oder stabil zu halten, bestehen in der Organisation von Medienveranstaltungen. In diesen kann zu Veränderungen im Pandemiegeschehen oder zu Entscheidungen über neue Maßnahmen Stellung genommen werden oder es können relevante, neue Forschungserkenntnisse laienverständlich eingeordnet werden [24]. Insbesondere der Effekt für Bildungsunterschiede weist darauf hin, dass eine Einordnung und Übersetzung der rapide wachsenden Forschungsergebnisse in einfache Sprache wichtig sind. Wenn Ergebnisse aus Bevölkerungsumfragen wie COSMO in der Öffentlichkeitsarbeit der Organisationen und ggf. auch Ministerien akzentuiert und zur Entwicklung und Begründung von Maßnahmen verwendet werden, betont dies die Tatsache, dass die Bedürfnisse der Bevölkerung, insbesondere auch der Zielgruppen mit geringerem Vertrauen, für Institutionen in der Pandemie handlungsleitend sind. Außerdem sollten Entscheidungen unter Unsicherheit als solche kommuniziert werden [5].

In Hinblick auf die Nutzung sozialer Medien als Kommunikationskanal sollte in zukünftiger Forschung zu Vertrauen in Institutionen untersucht werden, ob die Zielgruppe mit geringem Vertrauen in die Institutionen auf einschlägigen Kanälen wie Messengerdiensten (z. B. Telegram, WhatsApp) erreicht werden kann. Eine genaue Untersuchung ist vorher jedoch dringend angeraten, um Bumerangeffekte [25] auszuschließen.

Die hier präsentierten Forschungsergebnisse decken sich mit anderen Umfragen im deutschsprachigen Raum zu Vertrauen in der Pandemiebewältigung. In einem Policy-Paper der Universität Konstanz wird zum Beispiel berichtet, dass Probanden durchschnittlich ein mittleres bis hohes Vertrauen in die effiziente Krisenbewältigung haben (im Durchschnitt 6 von 10 möglichen Punkten) und überzeugt sind, dass alle Patienten gleich behandelt werden (im Durchschnitt 6,8 von 10 möglichen Punkten; [26]).

### Limitationen

Die Befragung wird querschnittlich erhoben, daher können keine Aussagen zu Kausalzusammenhängen oder individuellen Entwicklungen getroffen werden. Es wurden keine Ergebnisse zur Bekanntheit der Institutionen gesammelt, bevor Vertrauen erhoben wurde. Allerdings gab es in der Abfrage von Vertrauen die Option, „keine Angabe möglich“ auszuwählen. Der Prozentsatz der Befragten, die keine Angabe machen konnten, lag für das RKI zwischen 2 % und 3 %, für die BZgA zwischen 7 % und 10 %. Alle Daten sind Selbstauskünfte, daher ist nicht ausgeschlossen, dass insbesondere Angaben zu Verhaltensweisen geringen Verzerrungen durch soziale Erwünschtheit unterliegen. Das Potenzial sozial erwünschter Angaben ist jedoch in Onlinestudien generell geringer ausgeprägt als in Telefoninterviews oder persönlichen Befragungen [27]. Die Stichprobe ist hinsichtlich der Bildung nicht repräsentativ, es nehmen eher höher Gebildete an der Befragung teil. Aus den Regressionen zum Ursprung von Vertrauen zeigt sich jedoch, dass demografische Variablen insgesamt nur einen geringen Erklärungswert für das Vertrauen in RKI und BZgA besitzen.

## Schlussfolgerung

Vertrauen über die ersten 6 Monate der Pandemie ist hoch und stabil, dies wiederum steht in positivem Zusammenhang mit pandemierelevantem Verhalten sowie hoher Akzeptanz der Maßnahmen. Im weiteren Verlauf der Pandemie kann damit eine gute Grundlage für zielgruppenspezifische Information von RKI und BZgA vorausgesetzt werden. Weniger als demografische Unterschiede sind hierbei Zielgruppen zu adressieren, die sich in ihrer affektiven Risikowahrnehmung unterscheiden. Besonders solange zur Eindämmung der Pandemie ausschließlich Verhalten und Restriktionen zur Verfügung stehen und noch nicht auf wirksame therapeutische Interventionen zurückgegriffen werden kann, sollte Vertrauen in die Institutionen sowie Akzeptanz von Verhalten und Restriktionen regelmäßig überprüft und ggf. gefördert werden. Die Ergebnisse zeigen eine hohe Akzeptanz und Vertrauen in die Institutionen. Diese Haltung kann auch für die Vermittlung weiterer notwendiger Maßnahmen genutzt werden.
